# A Systematic Review of Spironolactone Nano-Formulations for Topical Treatment of Skin Hyperandrogenic Disorders and Chronic Wounds

**DOI:** 10.3390/pharmaceutics17010027

**Published:** 2024-12-27

**Authors:** Saedah Dereiah, Muhammad Usman Ghori, Barbara R. Conway

**Affiliations:** 1 Department of Pharmacy, University of Huddersfield, Huddersfield HD1 3DH, UK; saedah.dereiah@hud.ac.uk (S.D.); m.ghori@hud.ac.uk (M.U.G.); 2 Institute of Skin Integrity and Infection Prevention, University of Huddersfield, Huddersfield HD1 3DH, UK

**Keywords:** spironolactone, nano-formulations, skin, acne, wounds, transdermal drug delivery, drug permeation

## Abstract

**Background/Objectives:** Spironolactone (SP), an aldosterone inhibitor widely used to treat androgen-dependent disorders such as acne, hirsutism, and alopecia, has demonstrated therapeutic potential in both oral and topical formulations. However, SP’s low solubility and poor bioavailability in conventional formulations have driven the development of novel nanocarriers to enhance its efficacy. This review systematically examines recent advancements in SP-loaded nanocarriers, including lipid nanoparticles (LNPs), vesicular nanoparticles (VNPs), polymeric nanoparticles (PNPs), and nanofibers (NFs). **Methods:** A search strategy was developed, and the relevant literature was systematically searched using databases such as Scopus, PubMed, and Google Scholar. The review process, including screening, inclusion, and exclusion criteria, adhered to the Preferred Reporting Items for Systematic Reviews and Meta-Analyses (PRISMA) guidelines. **Results:** A comprehensive analysis of 13 eligible research articles, corresponding to 15 studies, highlights key aspects such as encapsulation efficiency, stability, particle size, and in vitro and in vivo efficacy. Six studies focused on lipid nanoparticles (LNPs), including solid lipid nanoparticles (SLNs) and nanostructured lipid carriers (NLCs), which were found to improve SP’s bioavailability and skin permeation. Another six studies investigated vesicular nanoparticles (VNPs), such as ethosomes and niosomes, demonstrating superior skin targeting and penetration capabilities. Two studies on polymeric nanoparticles (PNPs) showed effectiveness in delivering SP to hair follicles for the treatment of alopecia and acne. Additionally, one study on SP-loaded nanofibers indicated significant potential for topical rosacea therapy. **Conclusions:** SP-loaded nanocarrier systems represent promising advancements in targeted topical therapy. However, further clinical studies are required to optimize their safety, efficacy, and delivery mechanisms.

## 1. Introduction

Spironolactone (SP) is an aldosterone inhibitor approved more than sixty years ago for the treatment of hypertension as a fourth-line therapy, as well as for oedema and hyperaldosteronism [1,2]. Due to its ability to act as an androgen blocker, SP is commonly used to treat hyperandrogenic skin disorders such as acne, hirsutism, and alopecia, particularly in women with polycystic ovary syndrome (PCOS). It is generally used as a second-line therapy for PCOS, but for sexually inactive women who do not take oral contraceptives, it can be used as a first-line treatment [2,3,4]. A retrospective study assessed the efficacy of oral SP (100 mg) as a first-line therapy for hyperandrogenic skin diseases following a 6-month treatment period. Despite the discontinuation of treatment many months earlier, the study confirmed the effectiveness and safety of SP for treating acne, hirsutism, and alopecia [5]. Spironolactone prescriptions for dermatological disorders, particularly acne and hirsutism, have increased significantly in recent years. From 2017 to 2020, prescriptions rose three- to four-fold, with spironolactone being prescribed at similar rates to oral antibiotics by both dermatologists and non-dermatology providers [6]. The shift reflects a growing preference for non-antibiotic treatments, although concerns about its off-label status, dosing uncertainties, and potential side effects like hyperkalemia remain barriers to broader adoption. Despite these challenges, spironolactone remains a favored option, often used in combination with other treatments like oral contraceptives [7].

Additionally, spironolactone (SP) functions as an inhibitor of mineralocorticoid receptors (MRs) and exhibits anti-inflammatory activity [2]. Some studies have reported that MRs are expressed in human skin epidermis, hair follicles, sebaceous units, and sweat glands [8,9,10]. Since MRs can be activated by glucocorticoids, the use of topical glucocorticoids can lead to wound healing failure, skin atrophy [8], skin fragility, dehydration, injuries, accelerated aging, and increased infection risk [11]. The MR antagonist has been shown to enhance wound healing by targeting sodium channels in the epithelium, promoting keratinocyte re-epithelialization, proliferation, and differentiation [11,12]. Topical SP has also been found to increase ceramide and cholesterol levels in skin treated with clobetasol propionate [10]. In this context, the co-administration of topical SP with the glucocorticoid dexamethasone has been proposed to explore SP’s potential to counteract the wound-healing impairment caused by glucocorticoids in the cornea [13]. Preclinical tests on rabbits demonstrated a significant improvement in wound closure in the group treated with SP compared to the control group, confirming SP’s efficacy in counteracting wound healing abnormalities caused by glucocorticoids and its competitive ability to block MRs [9]. Another clinical study performed in 2023 on rats reported that 0.1% SP eyedrops accelerated wound healing in rat corneas, reduced inflammation, improved epithelial integrity, enhanced nerve repair, and supported equilibrium reconstruction of the corneas [14].

However, it has been reported that oral SP can cause significant side effects, including abdominal pain, fatigue, depression, headache, menstrual irregularities, male embryo feminization, increased urine excretion, and hyperkalemia [3,15,16,17]. To mitigate these common side effects while maintaining therapeutic effectiveness, topical delivery approaches have become more attractive and have been explored in various clinical studies. For example, Ayatollahi and Samadi [18] demonstrated the efficacy and safety of a topical 5% SP cream for treating acne, while Abdel-Raouf and Aly [19] reported the effectiveness of a topical 1% SP gel for treating alopecia in both males and females, either as a standalone treatment or in combination with 5% minoxidil gel. Another study suggested that topical SP could potentially be used to reduce the symptoms of androgenic-related rosacea [20]. Topical SP has generally been found to be safer than oral SP for both genders [17,21].

SP has several drawbacks, including low aqueous solubility and poor oral bioavailability due to its hydrophobic nature. Various formulation strategies have been employed to improve the solubility and bioavailability of poorly water-soluble molecules, such as liposomes, lipid nanocarriers, nanoemulsions, cyclodextrin complexes, chitosan complexes, and cubosome nanoparticles [22]. The non-selective action of SP [21] is another challenge that has driven the development of targeted novel SP formulations. These limitations have motivated the development of SP nano-formulations in pharmaceutical research, aimed at targeting hyperandrogenic disorders and chronic wound-healing diseases while overcoming the shortcomings of conventional SP delivery. Therefore, the aim of this review was to screen, collate, and analyze the literature on SP topical nano-formulations, focusing on the encapsulation capacity, the composition optimization, and the carrier vehicles as primary outcomes. Additionally, this review will highlight the limitations of each nanocarrier system and discuss their advantages over conventional topical delivery systems as secondary outcomes of this systematic review.

## 2. Methodology

### 2.1. Search Plot, Information Sources, and Screening Process

The search strategy, following the PRISMA 2020 guidelines (Supplementary Materials) [23], incorporated distinct stages of identification, screening, eligibility, and inclusion. A systematic search was conducted to identify relevant research studies published from January 1996 to July 2023 since inception. An inclusive search plot based on Scopus, PubMed, and Google scholar databases was used. A search mesh based on the search terms (“spironolactone” OR “topical Spironolactone”) AND (“formulations” OR “acne” OR “acne vulgaris” OR “skin disease”) was employed. The titles and abstracts of the studies obtained were examined, and any studies that were not relevant to the scope of the present systematic review were excluded. The full texts of the remaining studies were carefully reviewed to assess their eligibility. Any additional studies that did not align with the rationale of the review were also removed.

### 2.2. Study Selection

For this systematic review, two reviewers independently assessed the suitability of the eligible records to ensure accuracy and reduce bias. Cohen’s Kappa was used to assess inter-rater reliability, providing a measure of agreement beyond chance, with values above 0.75 indicating good reliability [24]. Discrepancies between reviewers were resolved through discussion, and if consensus could not be reached, a third reviewer was consulted to adjudicate, where applicable.

### 2.3. Data Extraction and Collection

The information was extracted from all the eligible studies using a pre-defined template (Table 1), including type of nanocarrier, formulation composition, particle size, polydispersity index, zeta potential charge, entrapment efficiency, and manufacturing method. The extracted information was subsequently tabulated using Microsoft Word 2019 following an already published method [25,26,27,28,29,30,31,32]. The final extracted data were reviewed and validated for consistency and completeness, ensuring alignment with the study objectives and systematic review guidelines.

### 2.4. Risk of Bias Assessment

A recently developed framework [33], which relies on evaluating studies related to pharmaceutical formulations, was utilized to assess the potential bias in all the relevant studies. This framework encompasses six essential aspects, namely research rationale, description of the methodology, characterization and testing, description of results, description of discussion, and conclusions. Each study was individually evaluated using this framework by the research team to determine its risk of bias. The authors then conducted a thorough panel discussion to finalize their recommendations for each study based on their initial examination.

## 3. Results and Discussion

**Figure 1:**
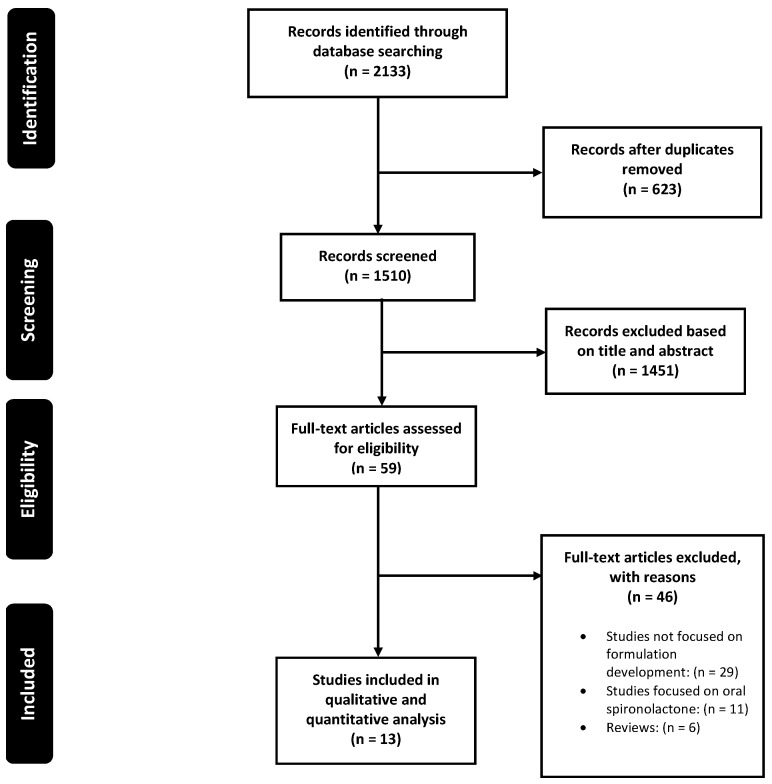
Search strategy for literature screening according to PRISMA guidelines [23].

This study initially identified 2133 articles from various database resources. After removing duplicates using Endnote, 1510 articles remained. A thorough screening of titles and abstracts was conducted, resulting in 59 articles. These were then subjected to a full-text analysis based on the required criteria, which narrowed the selection down to 25 articles. Further limitations were applied, ultimately leading to the inclusion of 13 articles, corresponding to 15 studies, in the systematic review [34,35,36,37,38,39,40,41,42,43,44,45,46]. The detailed screening process in accordance with the PRISMA guidelines is illustrated in Figure 1. In assessing the inter-rater reliability of the inclusion criteria, Cohen’s Kappa was calculated to measure the level of agreement between the two independent reviewers. The Kappa value was 0.94, indicating an excellent agreement between the raters [24]. This high value suggests that the raters had a very strong agreement on the classification of studies, with statistically non-significant disagreement.

**Figure 2:**
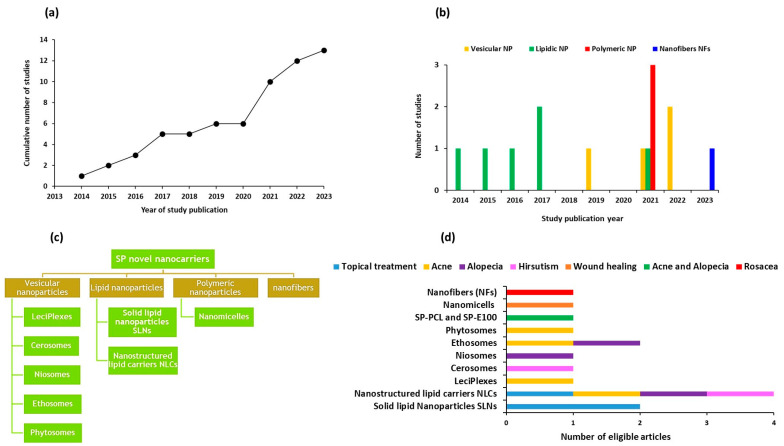
(**a**) Cumulative studies published on SP nanocarriers over the years, (**b**) studies published each year categorized by the type of nanocarrier, (**c**) classification of the various SP nanocarriers based on the current literature review, and (**d**) different nanoparticulate formulations and their clinical applications for various skin conditions.

The development of SP-based nanocarriers for topical delivery has seen significant growth since its emergence in 2014, as illustrated in Figure 2a. Based on the included studies, these nanocarriers encompass a wide range of systems, including vesicular nanoparticles (such as leciPlexes, cerosomes, niosomes, ethosomes, and phytosomes), lipid nanoparticles (solid lipid nanoparticles and nanostructured lipid carriers), polymeric nanoparticles, including nanomicelles, and nanofibers, as depicted in Figure 2b,c. These nanoparticulate systems have been applied for various skin conditions, including acne, alopecia, hirsutism, wound healing, and rosacea, as shown in Figure 2d. The increasing diversity and application of these nanocarriers highlight their potential for enhancing the therapeutic efficacy of SP through targeted delivery and improved skin penetration, making them promising candidates for addressing a broad range of dermatological conditions.

**Figure 3:**
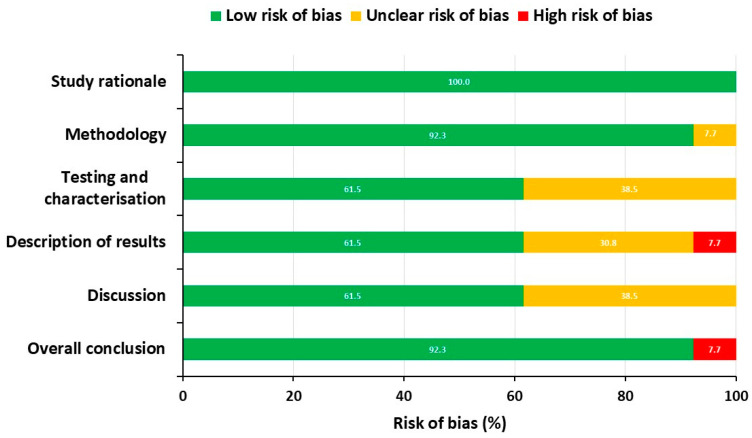
Overall assessment of risk of bias across six domains covered in the eligible studies.

The distribution of risk of bias across the included studies is shown in Figure 3. Overall, the studies demonstrated a generally low risk of bias. However, some areas of concern were noted, particularly regarding unclear bias in several key aspects. Specifically, 7.7% of the studies had ambiguous methodologies, while 38.5% showed unclear bias in testing and characterization procedures. Similarly, 38.8% of the studies lacked clarity in their description of results, and 38.5% exhibited unclear bias in the discussion sections. In addition to these concerns, the framework identified instances of high bias in specific domains. For example, 7.7% of the studies entirely omitted critical details in their result descriptions, while another 7.7% showed bias in their overall conclusions. These gaps suggest a need for more rigorous reporting standards, particularly in ensuring the transparency and thoroughness of both the experimental results and the discussions of findings. Addressing these issues in future studies will help improve the reliability and reproducibility of research on SP nanocarriers.

### 3.1. Lipid Nanoparticles (LNPs)

#### Solid Lipid Nanoparticles (SLNs) and Nanostructured Lipid Carriers (NLCs)

**Figure 4:**
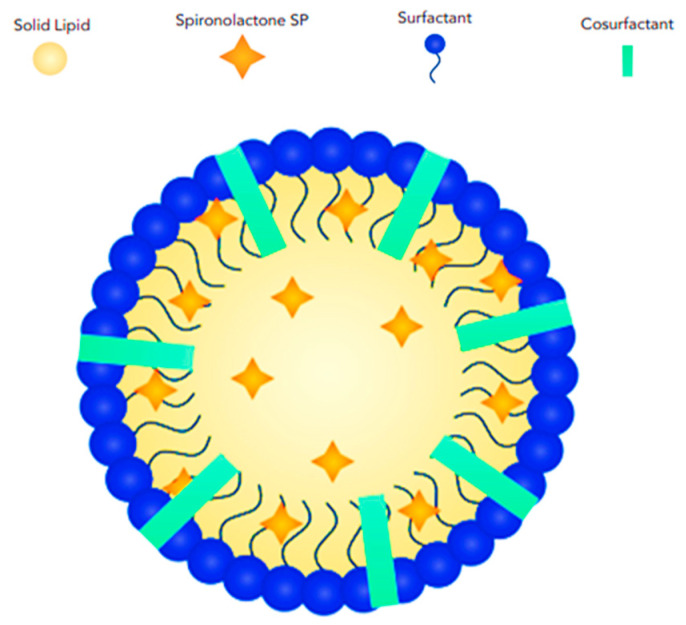
Schematic illustration showing the structural features and arrangement of SP-loaded solid lipid nanoparticles (SLNs).

Solid lipid nanoparticles (SLNs) are colloidal structures composed of biocompatible solid lipids, such as fatty acids, waxes, and triglycerides, combined with surfactants that stabilize the solid lipids within an aqueous medium [47,48,49]. SLNs typically have a core–shell structure, and the central part (core) of the nanoparticle is composed of the solid lipid. The drug molecules can be incorporated into the lipid matrix or adsorbed onto its surface. Surrounding the core is a thin layer of surfactant molecules. This shell provides stability to the nanoparticle, prevents aggregation, and controls the release of the drug (Figure 4).

**Figure 5:**
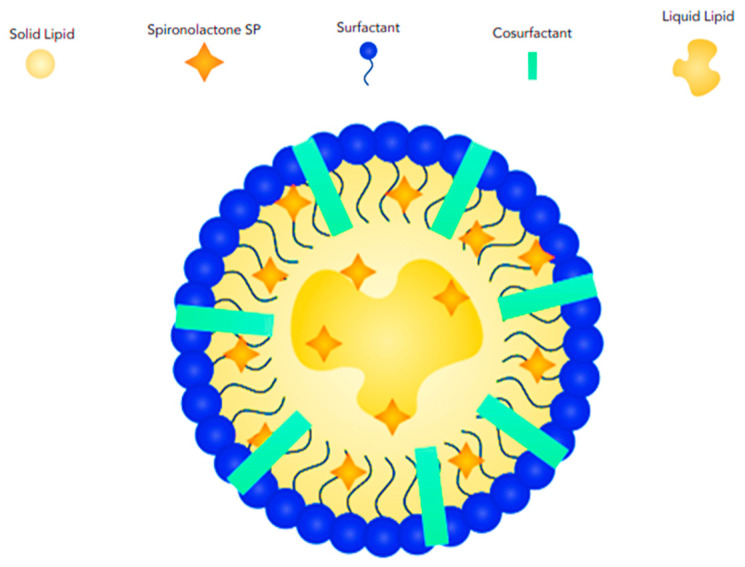
Schematic illustration showing the structural features and arrangement of SP-loaded nanostructured lipid carriers (NLCs).

SLNs were introduced in the 1990s by replacing the oil components in emulsions with fatty acids, resulting in the formation of solid nanoparticles (NPs) at room and skin temperatures [50]. While SLNs are highly stable carriers, capable of incorporating both lipophilic and hydrophilic drugs and protecting unstable drug molecules from chemical degradation [51], they have some drawbacks. These include drug leakage from NPs during storage, limited drug delivery and deposition in the skin [52,53], decreased drug flux to the skin due to the high viscosity of NP formulations, the presence of lipid polymorphic forms, and a tendency to agglomerate [53]. Nanostructured lipid carriers (NLCs) are an advancement of lipid nanoparticles (NPs) and are composed of a mixture of non-toxic solid lipids (up to 70%) and liquid lipids (up to 30%) with an emulsifier or co-emulsifier. The particle size (PS) of NLCs ranges from 50 nm to 500 nm [54,55]. Liquid lipids play a critical role in enhancing the stability of nanocarriers by limiting lipid matrix recrystallization, increasing lipophilic drug loading, reducing drug leakage from NPs, and improving drug release [56,57]. NLCs are categorized into three types: imperfect crystal, amorphous, and multiple oil-in-fat-in-water, depending on the lipid composition and the solid/liquid ratio. NLCs often exhibit a more complex structure compared to SLNs due to the presence of both solid and liquid lipids. The structural arrangement can vary depending on the specific composition and processing conditions. However, a common model involves a core–shell structure; the core may contain a mixture of solid and liquid lipids, with the drug molecules dispersed or encapsulated within the lipid matrix. The shell is composed of surfactant molecules, providing stability and controlling drug release (Figure 5). NLCs have advantages over SLNs, such as greater flexibility in modifying drug release by altering the type or amount of emulsifier or oil [58]; they also have drawbacks, including potential skin irritation due to the surfactants used [58,59].

SP was successfully formulated as SLNs by Kelidari, Saeedi [34] using both emulsification and ultra-sonication methods. The SLNs were optimized by screening different surfactant types and adjusting the surfactant-to-drug-lipid concentration. The SP/lipid ratio was 1:4. Later, Kelidari, Saeedi [35] conducted a comparative study between SP-loaded SLNs and SP-loaded nanostructured lipid carriers (NLCs) to investigate their disintegration rates and stability. The initial step involved assessing the solubility of SP in four types of solid lipids blended with oleic acid at different ratios to determine the most suitable lipid carrier for SP. Stearic acid (SA) was selected as the optimal solid lipid for SP because it created a homogeneous suspension with SP after the heating process. After six months of storage at 4, 25, and 40 °C, the NPs were found to be more stable at 4 °C or 25 °C than those stored at 40 °C for both NP types. In vitro studies revealed that NLCs produced a faster drug release compared to SLNs, likely due to the presence of oleic acid in the NLC formulations.

Stearic acid (SA), a naturally occurring solid lipid with several clinical benefits, such as anti-inflammatory and antimicrobial properties, was chosen as the primary solid fatty acid for SLN manufacturing [60]. SA was the main lipid used in the SLN and NLC systems designed by Kelidari, Saeedi [34,35,36]. The entrapment efficiency (EE) increased from 59.85% to 77.1% when the SA amount increased from 0.05 g to 6 g in the SP-SLN formulation, with a corresponding increase in PS. Similar observations were reported for oxiconazole nitrate-loaded SLNs and piroxicam-loaded SLNs [61,62]. For oxiconazole nitrate-loaded SLNs, the EE increased by 13.65% when the SA concentration increased from 1% to 3% *w*/*w* [61], while for piroxicam-loaded SLNs, the EE improved by 14.1% when the SA concentration increased from 1% to 1.5% [62]. Moreover, increasing the SA content in the SP-SLN formulations from 0.05 g to 6 g enhanced the ability of SLNs to encapsulate higher amounts of SP, ranging from 12.5 mg to 400 mg, supporting the conclusion that SLNs could be a promising carrier for SP.

Kelidari, Saeedi [36] conducted a clinical trial comparing the efficacy of an SP-NLC-based gel with a conventional SP alcoholic gel in patients with acne vulgaris (AV). They found significant improvements in treating non-inflammatory lesions after two months of treatment for both formulations, with a notable increase in skin water content achieved by the SP-NLC gel. SA and oleic acid were the main components of the SP-NLC lipid mixture. The SP-NLC gel particles had a lower polydispersity index (PDI) and smaller PS compared to the SP alcoholic gel. In another study, Shamma et al. [37] aimed to use NLCs for hair follicle-targeted SP delivery to enhance the efficacy and safety of SP in treating alopecia. An interesting aspect of the study was the use of a full factorial design to optimize and assess the influence of different parameters on the SP-NLC formulation, followed by the creation of a polynomial equation to link factors and responses. After 24 h of in vitro drug release, the percentage of drug released from the optimized SP-NLCs (66%) was higher than that from a suspension (40%). Compritol 888 ATO (glyceryl behenate) was chosen as the primary solid lipid due to its low cytotoxicity and its ability to solubilize SP and other lipophilic drugs [37,63]. However, Compritol 888 ATO was later replaced by stearic acid in all subsequent SP-NLC formulations. Ramkar and Suresh [63] used Compritol 888 ATO as the solid lipid to produce finasteride NLCs for treating androgenic alopecia; EE was 84% with a PS of 379.8 nm [63], which is comparable to the outcomes reported by Shamma et al. [37]. Therefore, Compritol 888 ATO could be a suitable candidate solid lipid for preparing SP-loaded NLCs.

Shamma et al. [37] also indicated that Tween 80 and Transcutol-P improved dissolution, with the dissolution rate of NPs being higher than that of plain SP suspension due to their larger surface area [64]. Transcutol-P has a long history of use as a co-surfactant and a penetration enhancer in cosmetic and topically applied products. Additionally, a fibroblast toxicity test demonstrated the safety of Transcutol-P [65]. Amer et al. [38] performed a comparative assessment between SP-NLCs and progesterone (PG)-NLC formulations to evaluate their efficacy in treating hirsutism. Two hydrogels containing the selected SP-NLC and PG-NLC formulations were prepared by combining the nanocarrier dispersions with Carbopol (1% *w*/*v*) as a gelling agent. The final concentrations were 2% for SP and 8% for PG in the nanogels. In an ex vivo permeation study under identical conditions, SP-NLCs released 85% of SP compared to 45% from the control suspension after 24 h, while PG-NLCs showed higher drug release than the plain PG suspension. Furthermore, following the topical application of the nanogels, the SP-NLC hydrogel demonstrated better results in treating hirsutism over three weeks compared to the PG-NLC hydrogel. However, the systemic therapeutic effects of both nanogels were analyzed and confirmed by assessing a series of hormones and inflammatory mediators in rat serum, while the topical effects were not assessed.

Poloxamer 188, a non-ionic surfactant [66] approved for use in various formulations, including cutaneous, oral, liquid, and semisolid [67], was incorporated into NLC formulations by Amer et al. [38] to enhance their stability through steric hindrance stabilization. The optimized NLC carriers were formulated with oleic acid comprising 40% of the total lipid content. Moreover, increasing the proportion of SA in NLCs notably enhanced the carrier’s capacity to incorporate SP, aligning with findings from SP-loaded SLNs.

Overall, in vitro and in vivo tests demonstrated the benefits of SLNs and NLCs over conventional topical delivery systems and suggested that they are suitable and effective carriers for SP. This should encourage scientists to explore different concentrations of SP clinically to determine the effective therapeutic dose of SP at the nanotechnology level. The summarized characteristics of all the eligible studies’ fabricated SLNs and NLCs are presented in Table 2.

### 3.2. Vesicular Nanoparticles (VNPs)

#### 3.2.1. LeciPlexes (LPs)

**Figure 6:**
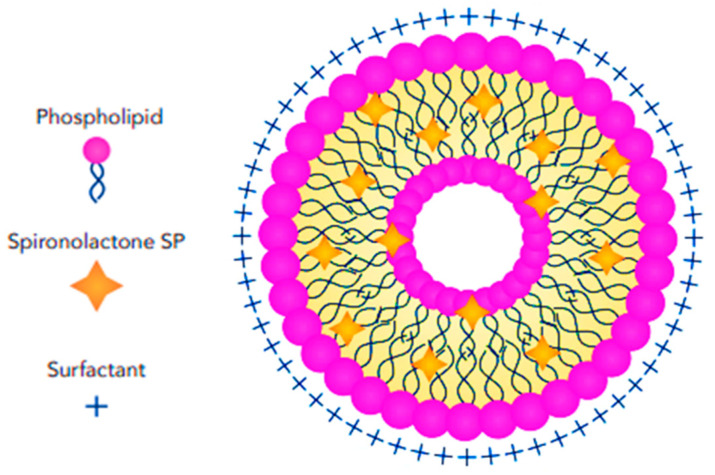
Schematic illustration showing the structural features and arrangement of SP-loaded LeciPlexes.

LeciPlexes (LPs) are a unique type of lipid-based nanoparticle that combine the advantages of liposomes and lipid nanoparticles. These are a specialized type of nanocarrier system, with phospholipids, cationic surfactants, and a biocompatible solvent being the main components developing a stable, versatile delivery platform. LPs offer improved biocompatibility and can enhance drug solubility. This outer layer is composed of phospholipids, like the cell membrane. It provides stability and controls drug release. LPs typically have a core–shell structure; the core is composed of the lecithin-based lipids, which can encapsulate the drug molecules. The outer shell is a phospholipid bilayer, providing protection and controlling drug release (Figure 6) [68,69].

LPs offer several advantages over conventional liposomes and other nanocarriers like SLNs, including a simple fabrication method and the use of biocompatible, non-toxic solvents that do not require removal from the formulation, unlike the organic solvents used in traditional liposome production. LPs were first reported in 2011, designed to enhance the anti-inflammatory and anti-cancer effects of quercetin following oral delivery [70,71]. A series of trials investigating LP nanocarriers with SP for targeting female acne was conducted by Salama et al. [39]. The study primarily examined the effects of SP concentration and the ratio of the cationic surfactant CTAB to lipid in the formulations. Although the PS of LPs increased significantly with higher drug concentrations (up to a certain point), PS decreased when the CTAB/lipid ratio increased. The drug encapsulation capacity of the nanocarriers increased from 85% to 93% as the CTAB ratio rose from 0.25:1 to 1.5:1. Similar findings were reported by Elmowafy et al. [72], who designed a vesicular system called EtholeciPlex to enhance the accumulation of minoxidil in rat skin following topical delivery. They highlighted two likely reasons for the slower release rate of the SP-LP gel compared to the suspension: the interaction between the drug and the CTAB, and the formation of a network gel that acts as a barrier, reducing drug release. However, the study did not explore the long-term stability of optimized nanoparticles nor assess their clinical effectiveness.

#### 3.2.2. Cerosomes

**Figure 7:**
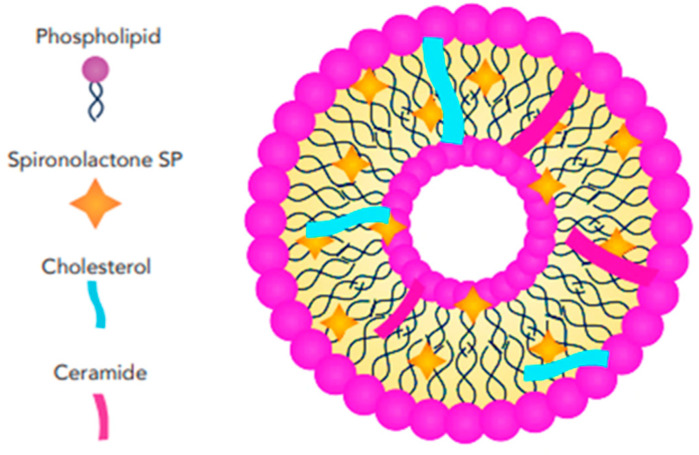
Schematic illustration showing the structural features and arrangement of SP-loaded ceresomes.

Cerosomes are a type of lipid-based nanoparticle used in drug delivery and various other biomedical applications. These nanoparticles are lipid vesicles that incorporate ceramides as a key structural component, which imparts unique properties when compared to conventional liposomes, typically composed of phospholipids, Figure 7. The primary advantages of cerosomes over common liposomes include their ability to dissolve in the outermost layer of the skin due to their sensitivity to keratin, as well as their specific localization in hair follicles. This makes them a promising candidate for targeting skin diseases and other dermatological treatments. In addition to their keratin sensitivity and follicular targeting, cerosomes offer enhanced stability compared to traditional liposomes. Ceramides, naturally present in the skin, provide structural rigidity, helping the cerosomes to resist degradation. This increases their potential to protect and deliver encapsulated therapeutic agents effectively. The stability of cerosomes makes them particularly suitable for applications that require sustained drug release or the transport of sensitive molecules, such as proteins, peptides, or nucleic acids, which might otherwise degrade rapidly in the body [73].

**Figure 8:**
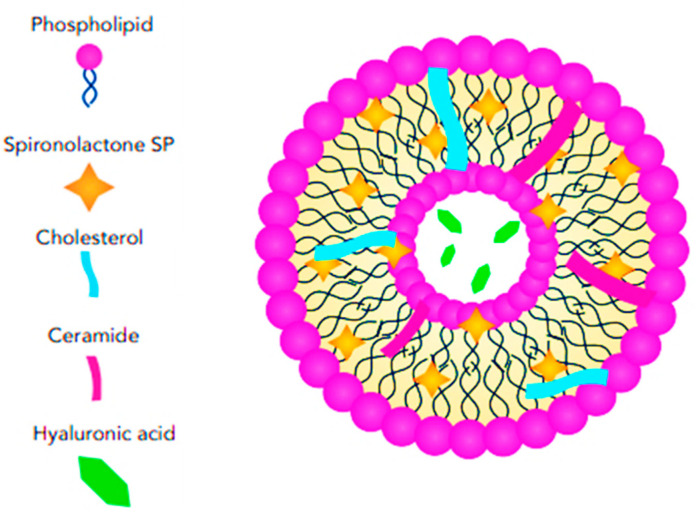
Schematic representation of SP-loaded HA-enriched ceresomes showing the structural features and arrangement.

Albash et al. [40] reported the potential for incorporating SP into a cerosome nanosystem strengthened with hyaluronic acid (HA) for the topical treatment of hirsutism (Figure 8). Several critical parameters affecting the vesicle properties were identified, and Design-Expert^®^ software (version 7.0) was used to design the preparation trials, reducing the number of trials required for SP-loaded hyaluronic acid-enriched cerosomes (HAECs). They fabricated the SP-loaded HAECs using an ethanol injection approach, combining SP, phosphatidylcholine (PC), ceramide, and either Kolliphor RH40 or Kolliphor EL as part of the lipid mixture, while HA was dissolved in water. Ceramides, which are one of the main components of the cell membrane, play a crucial role in the skin’s protective barrier and contribute to the stability of liposomes [73,74].

Albash and Fahmy [40] utilized ceramide for its hydration-enhancing and skin-protecting properties. Recent studies have shown that ceramide also functions as a permeability enhancer for hydrophilic drugs [75]. Kolliphor RH40 (polyoxyl 40 hydrogenated castor oil) is an edge activator surfactant that increases drug permeation and skin accumulation [40,76], while also reducing the aggregation of vesicles consisting of a lipid mixture [40]. According to the in-silico findings, molecular docking revealed the significant role of HA in enhancing the stability of the vesicles by increasing the binding sites between SP and PC. HA not only improves vesicle stability but also acts as a carrier, localizing SP within the PC bilayers. A study comparing the transdermal permeability properties of ketoprofen across different types of liposomes demonstrated that liposomes containing HA in their core exhibited greater drug flux and permeation coefficient values. Additionally, they have reported that all factors had a significant effect on EE%, with increasing concentrations of ceramide and HA leading to an increase in EE%, while Kolliphor RH40 produced better results than Kolliphor EL. The amount of ceramide was the only independent variable that negatively affected the PS of SP-loaded HAECs. The study reported an optimal formulation that remained stable after three months of storage testing and was deemed safe according to histopathological analysis [40]. However, it was not tested ex vivo or clinically.

#### 3.2.3. Niosomes and Ethosomes

**Figure 9:**
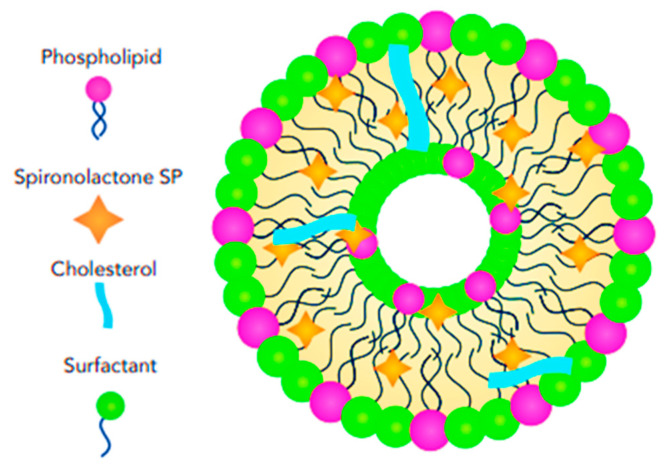
Schematic representation of SP-loaded niosomes showing the structural features and arrangement.

Niosomes are non-ionic surfactant vesicles similar to liposomes, but composed of non-ionic surfactants instead of phospholipids, offering potential advantages such as improved stability, reduced toxicity, and lower cost. Structurally, they feature a bilayer formed by non-ionic surfactants, which consist of a hydrophilic head group and a hydrophobic tail that arrange themselves in a double-layered configuration to create a stable vesicle, Figure 9. The core of the niosome can be either aqueous or oily, depending on the formulation, allowing for the encapsulation of various molecules such as drugs, proteins, and nucleic acids. Niosomes were first formulated in 1979 by Handjani-Vila et al. for use in cosmetics, specifically in moisturizing and tanning products [77]. Common surfactants used in niosome fabrication include Spans, Tweens, and Brij. Niosomes are more stable against oxidation than conventional liposomes, and they can incorporate both hydrophilic and hydrophobic drugs, with a more cost-effective production process [78,79]. In addition to niosomes, ethosomes have emerged as novel ethanol-enriched vesicular nanocarriers, composed of 2–5% phosphatidylcholine (PC), ethanol (20–45%), and water. Ethosomes enhance the permeation of a wide range of hydrophilic and lipophilic drugs, enabling both topical and systemic delivery, and increase drug deposition in the skin [80,81].

**Figure 10:**
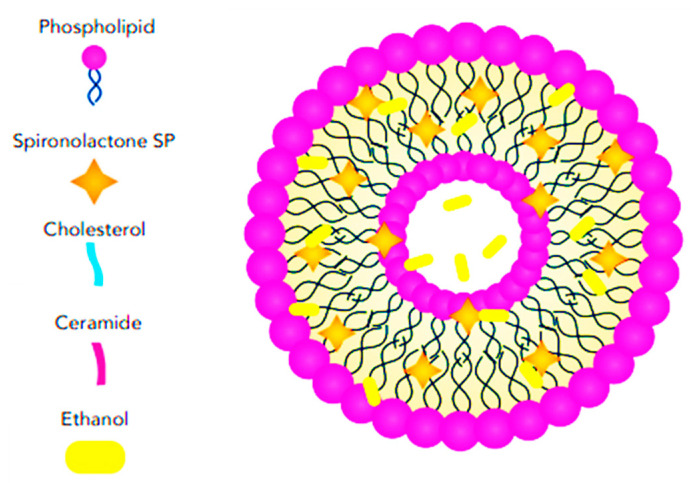
Schematic representation of SP-loaded ethosomes showing the structural features and arrangement.

However, ethosomes are vesicular carriers formed by phospholipids and ethanol, offering unique advantages in drug delivery due to the incorporation of ethanol. Structurally, ethosomes consist of a phospholipid bilayer, like liposomes, which forms a barrier capable of encapsulating various molecules. However, the defining feature of ethosomes is the presence of ethanol within both the bilayer and the aqueous core. Ethanol interacts with the phospholipids, altering their packing and increasing the fluidity of the bilayer, Figure 10. This results in ethosomes having a generally smaller size than liposomes, enhancing their ability to penetrate the skin and other tissues. The increased fluidity not only improves drug release but also facilitates greater cellular uptake. Moreover, ethosomes demonstrate enhanced skin penetration compared to traditional liposomes, making them particularly effective for transdermal drug delivery applications.

Abdallah et al. [41] reported two nanoparticle preparations with different doses of SP to compare and evaluate which vesicular nanocarrier—SP-loaded niosomes or SP-loaded ethosomes—was more stable and capable of encapsulating SP. Each formulation was treated as a separate study. Hydroxypropyl methylcellulose was used as a gelling agent to incorporate the optimized nano-formulations for ex vivo studies. The release rate of the encapsulated SP was similar for both nanocarriers, reaching 70% after one day during the diffusion test.

Abdallah et al. [41] developed SP-loaded niosomes using a thin-film hydration method, incorporating phosphatidylcholine (PC) and cholesterol as solid lipids and Span 40 as a stabilizer. The study identified and interpreted the effects of several essential variables, such as the ratio of SP to the lipid blend, lipid concentration, and the ratio of cholesterol to surfactant, on the formulation properties of the nanoparticles. Although the niosomes were successfully fabricated, they could not encapsulate more than 30 mg of SP, likely due to bilayer saturation. In a screening test, three non-ionic surfactants (Span 20, Span 40, and Span 80) were assessed for their ability to form SP-loaded niosomes. Span 40 was the most effective, producing stable and acceptable SP-loaded niosomes due to its high transition temperature (42 °C) and solid state at ambient temperature. For example, a comparative study using Span 40 and Span 60 to encapsulate the water-soluble drug salidroside found Span 40 to be more effective in increasing EE% and PS [82]. Moreover, Zhang et al. (2015) reported that Span 40 improved the permeability and accumulation of salidroside in the skin when encapsulated in niosomes [83].

SP-loaded ethosomes were successfully formulated by Abdallah et al. [34] using ethanol as a key component in concentrations ranging from 10% to 20% *w*/*w*. Ethanol contributes to NP stability and reduces PS due to its negative charge on the ethosomal surface, which prevents the formation of aggregated vesicular layers. However, increasing ethanol concentration beyond a certain limit reduced EE% and drug release. A separate study on retinyl palmitate-loaded ethosomes for treating skin diseases highlighted the role of ethanol in reducing NP size while increasing zeta potential (ZP) and EE% [84]. In contrast to niosomes, ethosomes successfully encapsulated a substantial amount of SP, up to 100 mg, with no sedimentation, and the PS of the optimal formulation was smaller, despite the higher SP content [41]. The paper also provides insights into the long-term stability of the nanocarriers, measuring sedimentation rates three months after production. Unlike other studies, Abdallah et al. [41] selected the optimized formulations based on PS alone, favoring sizes below 70 nm to achieve maximum delivery of SP to deeper skin layers.

Verma and Singh [42] also presented a topical gel containing SP-loaded ethosomes for treating acne, using ethanol and lipid concentrations ranging from 30% to 40% and 2% to 4%, respectively. The study concluded that ethanol concentrations exceeding 40% increased the likelihood of vesicle leakage. In vitro tests showed that the ethosome nanogel had 2.5 times greater permeation than the conventional gel, and the ethosomes demonstrated superiority over the plain gel in terms of both skin deposition and efficacy in treating acne.

#### 3.2.4. Phytosomes

**Figure 11:**
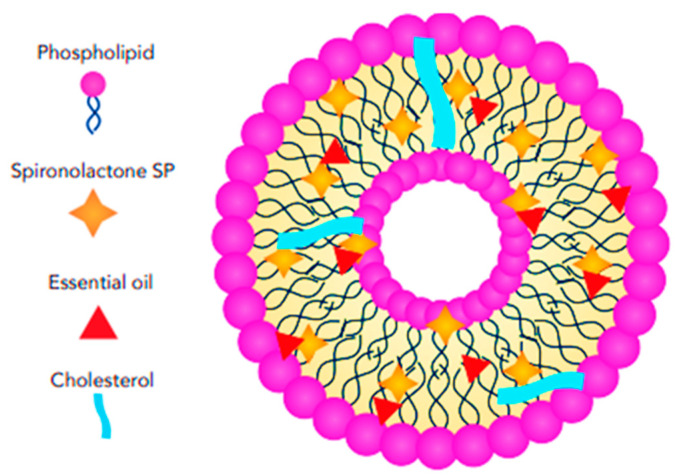
Schematic representation of SP-loaded phytosome showing the structural features and arrangement.

Phytosomes are NPs similar to liposomes in respect of their structural composition, containing PC, polyphenol extracts, and organic solvents. Phytosomes have a vesicular structure resembling small cells, with a phospholipid core primarily composed of phosphatidylcholine, which forms a lipid bilayer similar to cell membranes, enhancing interaction with biological tissues and facilitating absorption. The core encapsulates active phytoconstituents, such as flavonoids or alkaloids, protecting them from degradation and increasing stability. The phospholipids’ polar head group (choline moiety) interacts with the polar functionalities of the phytoconstituents, forming a stable complex, while the lipid-soluble tail interacts with the hydrophobic regions, further stabilizing the system [85]. The structural features of a phytosome are illustrated in Figure 11.

Over the years, various researchers have developed phytosomes to efficient topical drug delivery. For instance, Albash et al. [43] aimed to clinically evaluate the combined effect of SP and bergamot essential oil (BEO)-loaded phytosome nanocarriers in combating acne vulgaris (AV). The study employed a full factorial design to develop the experimental runs, and the nanocarriers were fabricated using a thin-film hydration approach. It was observed that increasing the concentration of the lipid phase (cholesterol and phosphatidylcholine) enhanced the EE%, negative charge, and PS of the nanocarriers, without significantly affecting the polydispersity index (PDI). Although the study did not include release or permeability assessments for the SP/BEO-loaded phytosomes, it provided valuable insight into their effectiveness by comparing the reductions in mild to moderate acne lesions after 8 weeks of treatment. SP/BEO-loaded nanosystems proved more effective than BEO-loaded phytosomes, despite the lower concentration of SP. However, it is important to note that the comparison was made using a split-face approach, which could introduce a high risk of bias, as patients were not blinded to the treatments. Additionally, the study did not evaluate the thermal stability of the nanoparticles, which is crucial for understanding their long-term stability and potential reactions between the components.

In summary, ethosomes encapsulated the highest amount of SP (100 mg) and had the smallest particle size (PS). LeciPlexes and niosomes significantly enhanced SP deposition in the skin layers, as demonstrated by ex vivo tests. In both in vitro and ex vivo studies, all VNPs outperformed the control systems. The summarized characteristics of all eligible studies that fabricated VNPs are presented in Table 3.

### 3.3. Polymeric Nanoparticles (PNPs)

Polymeric nanoparticles (PNPs) are nanocarriers with particle sizes ranging from 1 to 1000 nm that can encapsulate therapeutic molecules either within their core or dispersed throughout their matrix and surface. Polymeric nanoparticles can exhibit diverse structural arrangements, influenced by the polymer type, fabrication method, and desired properties [86,87]. These arrangements include solid cores (homogeneous or heterogeneous), hollow shells (single- or multi-layer), multi-core structures, micelle-like assemblies, vesicle-like formations, and dendritic structures. The choice of arrangement depends on factors such as drug loading, release kinetics, and targeting specificity, making polymeric nanoparticles a versatile platform for various applications. Various polymers are used to produce these nanoparticles; however, when selecting polymers for polymeric nanoparticles, key considerations include biocompatibility and biodegradability for safety, compatibility with spironolactone to ensure effective encapsulation and protection, and suitability for fabrication methods like nanoprecipitation. Robust mechanical properties maintain nanoparticle integrity, while controlled drug release ensures sustained therapeutic effects. Polymers with modifiable functional groups, such as polyethylene glycol, enable surface enhancements for stability and targeting. Prior regulatory approval of polymers expedites clinical translation, with examples like poly(lactic-co-glycolic acid) (PLGA), poly-ε-caprolactone (PCL), and chitosan widely used for pharmaceutical applications [88,89,90].

A recent comparative study reported on two types of polymeric NPs for SP follicular skin targeting and their compatibility with SP, stability, and ability to establish a safe and effective treatment for alopecia and acne [44]. The first polymer was PCL, a synthetic polymer commonly used in pharmaceutical applications due to its biodegradability and non-toxicity [91,92]. The second type of NP was made using EL100 polymer, a copolymer of methacrylic acid/methyl methacrylate. The study focused on follicular targeting of SP and the required particle sizes of nanocarriers to achieve this, utilizing three different sizes of NPs, all larger than 100 nm: 180.0 ± 1.6 nm and 126.8 ± 1.0 nm for PCL nanocarriers, and 102.7 ± 7.1 nm for EL100 nanocarriers. Differential scanning calorimetry (DSC) and thermogravimetric analysis (TGA) were used to establish compatibility. Despite the thermal analysis showing a significant decrease in degradation temperature for the mixture (130 °C) compared to SP (237 °C), it was argued that this would not affect the formulation of nanostructures produced at temperatures lower than the determined decomposition temperature. Additionally, the mass loss was below 10% for most excipients, which was considered acceptable by the authors. Furthermore, stability tests over three months at storage temperatures of 25 °C and 6 °C revealed no significant changes in the three prepared NPs in terms of PS, PDI, ZP, and EE%. The study also examined the irritation potential of the NPs using the HET-CAM test, which confirmed the safety of all the nano-formulations for topical therapy. In vitro drug release tests in buffered water and oleic oil found that after 8 h, the drug was released faster into the oil than the aqueous media for all types of NPs. The authors concluded that PLC-180, with the largest PS, had the highest ability to accumulate SP in hair follicles compared to the smaller sizes (126 nm and 102 nm), likely due to its higher EE%. An equation was used to measure a follicular targeting factor for each type of NP, representing the ratio of drug deposited in the hair follicle to the overall percentage of drug diffused into the skin layers. The study successfully established a relationship between PS and the penetration efficacy of NPs, highlighting a novel and promising carrier for follicular targeting. However, further characterization and clinical trials involving the NPs are essential.

Dahmana at al. [45] reported the formation of nanomicelles using the polymer methoxy-poly(ethylene glycol)-di-hexyl-substituted-poly(lactic acid) (mPEGPLA) to increase the solubility of SP in water and to explore SP localization in pilosebaceous units (PSUs) to inhibit MR activation by glucocorticoid therapy and improve wound closure. mPEGPLA is an amphiphilic, biodegradable, and non-toxic copolymer used as a micelle carrier for poorly water-soluble drugs [93]. The determination of MR sites in porcine skin layers was accurately assessed using immunofluorescent labeling. The results showed the highest intensity of MR sites in hair follicles and sweat glands. To quantify SP and its metabolites, a UHPLC-ESI-MS method was applied, one of the critical steps in the study. The SP quantity in PSU was five times higher than in skin without PSU. However, in contrast to earlier findings, no evidence of SP metabolites was detected in the skin layers 12 h after topical application of the nanogel system. The study concluded that canrenone was significantly quantified and accumulated in porcine skin PSUs two-fold more than in skin without PSUs. Unlike other studies focusing on improving SP’s dissolution rate by masking its lipophilicity, this study aimed to enhance SP’s water solubility by over 200 times to improve its pharmacokinetics and accumulation in PSUs. The PS in this study was 21 nm, differing from Dahmana at al. [45], who found that the optimal PS for targeting hair follicles was over 100 nm. However, it agrees with Abdallah et al. [41], who recommended that ethosomes with a PS of 70 nm were best suited. This discrepancy could be due to different skin types used in the investigations and the type of NP components used, which affect each nanocarrier’s features, making it crucial to determine PS’s effect on permeability. A study by Ghasemiyeh at al. [94] determined that 300 nm NLC-loaded cyproterone acetate could be an ideal size to deliver the drug topically and specifically target hair follicles and PSUs. One critical requirement for developing novel, effective SP nanocarriers is determining which PS is optimal for SP follicular targeting.

Overall, PNPs could be a potential carrier for SP, but further studies are needed to incorporate higher amounts of SP, as all nanocarriers tested only encapsulated 10 mg of SP. The summarized characteristics of all the eligible studies fabricated PNPs are presented in Table 4.

### 3.4. Nanofibers

Nanofibers (NFs) are fibrous nanostructures that can be created from various materials, including natural and synthetic polymers, semiconducting substances, and carbon-based materials [95,96,97]. NFs are produced through different methods, such as drawing, phase separation, and electrospinning—the most commonly used method for creating nanofibers—among others [98,99]. Recently, Elhesaisy, Swidan [46] have developed SP-loaded nanofibers using polyvinylpyrrolidone (PVP) for the topical treatment of rosacea, aiming to avoid friction and scratching. The electrospinning method was employed for producing SP-NFs due to its ability to quickly convert the drug into an amorphous state, preventing crystallization. The amorphous nature of SP in the NFs was confirmed by X-ray diffraction and differential scanning calorimetry. To assess the effectiveness of SP in treating rosacea, croton oil was applied to rat ear skin to induce symptoms similar to rosacea. The results indicated the significant efficacy of SP-loaded PVP NFs in reducing rosacea erythema, outperforming conventional SP gel formulations. Safety and stability tests for SP-NFs were also conducted and confirmed, supporting the authors’ conclusion, that NFs could be a promising carrier for the topical delivery of SP.

## 4. Recent Advances and Future Prospects

Recent advances in nanotechnology have significantly improved the delivery of poorly soluble drugs, addressing a major challenge in pharmaceutical development. Nanoparticles (NPs) offer a versatile platform that enhances the solubility, stability, and bioavailability of hydrophobic drugs while minimizing adverse effects [100,101,102,103,104,105,106]. For example, the limited aqueous solubility of spironolactone often results in erratic bioavailability and suboptimal therapeutic outcomes [107,108,109]. Nanoparticles, including polymeric nanoparticles, lipid-based carriers, and nanocrystals, enhance solubility and dissolution rates by significantly increasing the surface area of the drug exposed to the dissolution medium. Lipid nanoparticles, such as solid lipid nanoparticles (SLNs) and nanostructured lipid carriers (NLCs), have demonstrated exceptional potential by combining high drug-loading capacities, stability, and biocompatibility, making them suitable for systemic delivery of hydrophobic drugs like spironolactone [110]. Nanoparticles also allow for controlled release and tissue-specific targeting, reducing dosing frequency and mitigating adverse effects such as hyperkalemia, commonly associated with spironolactone therapy [111]. Functionalization of nanoparticles with targeting ligands, such as peptides or antibodies, enables selective drug delivery to specific tissues, such as renal cells or myocardial tissues, further reducing off-target effects [112]. Advances in bio-responsive nanoparticles, which release their payload in response to specific physiological stimuli like pH or enzymatic activity, have shown promise in improving drug efficacy while minimizing systemic toxicity [113]. Despite these strides, challenges such as large-scale production, regulatory hurdles, and understanding long-term safety persist. Addressing these issues will be critical for translating these technologies from bench to bedside. Future research should focus on improving the scalability and reproducibility of nanoparticle formulations for clinical translation. Studies on the long-term safety, biodistribution, and immunogenicity of nanoparticles are critical for their successful application. Additionally, the development of hybrid nanoparticles combining organic and inorganic materials could offer enhanced multifunctionality, such as imaging-guided drug delivery. Exploration of patient-specific nanoparticle formulations tailored using advanced computational and machine learning models could pave the way for personalized therapies. By addressing these areas, nanoparticle-based systems could revolutionize the treatment landscape for poorly soluble drugs like spironolactone.

## 5. Conclusions

The studies reviewed provide compelling evidence supporting the potential of nanocarriers in enhancing the topical delivery of spironolactone for various skin conditions. Lipid-based carriers, such as SLNs and NLCs, showed improved stability and encapsulation efficiency while overcoming the solubility challenges of SP. These systems demonstrated significant improvements in drug release and bioavailability compared to conventional formulations. Vesicular nanoparticles, particularly ethosomes and niosomes, offered effective transdermal delivery by enhancing drug penetration and targeting specific skin structures. PNPs were shown to be highly effective in targeting hair follicles, making them a promising tool for treating conditions like alopecia and acne. Nanofibers, meanwhile, demonstrated excellent therapeutic potential for rosacea, with superior performance over traditional SP formulations. However, despite these advancements, further research, including clinical trials, is essential to establish the optimal particle size, encapsulation efficiency, and safety of SP-loaded nanocarriers. Future work should focus on addressing these gaps to fully realize the clinical potential of these novel delivery systems for SP.

## Figures and Tables

**Table 1:** Form used for data extraction from eligible studies.

Nanocarrier	Composition	PS (nm), PDI, ZP (mV)	EE (%)	Method	Reference
					
					
					
					
					
					

**Table 2:** A summary of the characteristics of the SLNs and NLCs used in eligible studies.

Nanocarrier	Composition	PS (nm), PDI, ZP (mV) *	EE (%)	Method	Reference
SLNs	Stearic acid, Tween 80, Span 80 and Span 60	PS: 88.9 ± 5.3 PDI: 0.356 ± 0.058 ZP: −23.9 ± 3.1	59.85 ± 10.05	Emulsion-solvent evaporation and ultrasonication	[34]
SLNs	Stearic acid Span 80 and Tween 80	PS: 311.8 ± 19.0 PDI: 0.199 ± 0.030 ZP: −13.6 ± 2.6	77.10 ± 2.6	Probe-ultrasonication	[35]
NLCs	Stearic acid Oleic acid Span 80 and Tween 80	PS: 146.4 ± 15.2 PDI: 0.225 ± 0.012 ZP: −35.1 ± 3.0	90.6 ± 3.5	Probe-ultrasonication	[35]
NLCs	Stearic acid Oleic acid Span 80 and Tween 80	PS: 239.7 ± 99.48 PDI: 0.286 ± 0.051 ZP: −21.39 ± 0.051	77.10 ± 2.6	Probe-ultrasonication	[36]
NLCs	Compritol^®^888 ATO (glyceryl behenate) Olive oil Tween^®^ 80 Transcutol-P	PS: 215.6 ± 20.4 PDI: 0.877 ± 0.023 ZP: −18.7 ± 0.92	87.36 ± 3.34	Emulsion-solvent diffusion and evaporation method followed by ultrasonication	[37]
NLCs	Stearic acid Oleic oil Tween 80 and Poloxamer 188	PS: 225.92 ± 0.41 PDI: 0.428 ± 0.01 ZP: −36.5 ± 0.6	89.99 ± 0.45	Cold homogenization technique	[38]

* Data of optimized formulation (PS = particle size; PDI = polydispersity index; ZP = zeta potential).

**Table 3:** Summarized characteristics of the VNP’ eligible studies.

Nanocarrier	Composition	PS (nm), PDI, ZP (mV) *	EE (%)	Method	Reference
LeciPlexes	Soybean Lecithin Phospholipid (PL-90G), Cetyltrimethylammonium bromide (CTAB), Transcutol HP, and water	PS: 337.0 ± 11.1 PDI: 0.3 ZP: +49.3 ± 3.5	88.7 ± 3.4	One-step Production using continuous vortex.	[39]
SP-loaded HAECs	Phospholipid Ceramides Hyaluronic acid Kolliphor RH40 Kolliphor EL	PS: 261 nm PDI: 0.482 ± 0.07 ZP: −9.0 ± 1.1	89.3 ± 0.3	Ethanol injection	[40]
SP-loaded niosomes	Phospholipid Cholesterol, Span 40 (Sorbitan monopalmitate)/Chloroform	PS: 71.99 ± 9.04 PDI: 0.388 ± 0.02 ZP: −13.4 ± 0.02	95 ± 0.07	Thin-film hydration	[41]
SP-loaded ethosomes	Phospholipids Propylene glycol Cholesterol Ethanol	PS: 40.25 ± 8.15 PDI: 0.799 ± 0.02 ZP: −36.2 ± 5.00	96.9 ± 0.08	Hot method	[41]
SP-loaded ethosomes	Phospholipids (PC) Ethanol	PS: 66 ± 1.94 PDI: 0.185 ± 0.017 ZP: - **	40.31 ± 0.67	Ethanol injection	[42]
SP- and BEO-loaded nano-phytosomes.	Phospholipids (PC), Bergamot essential oil (BEO), Cholesterol	- **	- **	Thin film hydration method	[43]

* Data of optimized formulation (PS = particle size; PDI = polydispersity index; ZP = zeta potential), ** data not available.

**Table 4:** Summarized characteristics of the PNPs’ eligible studies.

Nanocarrier	Composition	PS (nm), PDI, ZP (mV) *	EE (%)	Method	Reference
SP-PLC	PLC 40 or 125 mg Acetone 25 mL Tween 80 135 mg PVA 5 mg	PS: 180 ± 1.6 nm PDI: 0.11 ± 0.03 ZP: −15.3 ± 1.3	87.6 ± 0.8	Nanoprecipitation solvent displacement	[38]
SP-E100	EL100 125 mg Ethanol Tween 80 135 mg PVA 5 mg	PS: 102.7 ± 7.0 PDI: 0.09 ± 0.01 ZP: −19.0 ± 1.2	41 ± 0.1	Nanoprecipitation solvent displacement	[38]
SP-loaded nanomicelles	Copolymer mPEGPLA Acetone Water	PS: 21 PDI: 0.2 ZP: −43	- **	Sonication and pressure reducing	[45]

* Data of optimized formulation (PS = particle size; PDI = polydispersity index; ZP = zeta potential), ** data not available.

## Supplementary Materials

The following supporting information can be downloaded at: https://www.mdpi.com/article/10.3390/pharmaceutics17010027/s1.
